# Serum Human Epididymis Protein-4 (HE4) - A novel Approach to Differentiate Malignant from benign Breast Tumors

**DOI:** 10.31557/APJCP.2021.22.8.2509

**Published:** 2021-08

**Authors:** K S S Sai Baba, Mohd Abdul Rehman, J Pradeep Kumar, Maira Fatima, G S N Raju, Shantveer G Uppin, Noorjahan Mohammed

**Affiliations:** 1 *Department of Biochemistry, Nizam’s Institute of Medical Sciences, Hyderabad, India. *; 2 *Department of General Surgery, Osmania General Hospital, Hyderabad, India. *; 3 *Department of Surgical Oncology, Nizam’s Institute of Medical Sciences, Hyderabad, India. *; 4 *Department of Pathology, Nizam’s Institute of Medical Sciences, Hyderabad, India. *

**Keywords:** HE4, CA 15-3, CEA, breast tumors, breast cancer

## Abstract

**Background:**

The lack of sensitivity and specificity of existing diagnostic markers like Carbohydrate Antigen 15-3(CA15-3) and Carcinoembryonic antigen (CEA) in breast cancer stimulates the search for new biomarkers to improve diagnostic sensitivity especially in differentiating benign and malignant breast tumors. Expression of Human epididymal protein 4 (HE4) has been demonstrated in ductal carcinoma of the breast tissue. So we tried to evaluate serum HE4 levels as diagnostic marker in breast cancer patients and to comparatively assess serum HE4, CEA and CA15-3 in breast tumor patients both benign and malignant.

**Methods:**

Total 90 female subjects were included in the study. We selected 30 breast cancer cases (Malignant group) and 30 benign breast lump cases (Benign group) based on histopathology report. And other 30 were age matched apparently healthy controls (Control group). HE4, CEA and CA15-3 were analysed in serum samples of all subjects by Electrochemiluminiscence immunoassay method.

**Results:**

A significant difference in the median (IQR) of HE4 (pmol/l) was identified among malignant, benign and control groups {62.4(52.6-73.7) vs 49.3(39.8-57.4) vs 52.3(50.6-63.3) P=0.0009} respectively. The cutoff value for prediction of breast cancer was determined at >54.5 pmol/l for HE4, with a sensitivity of 73.3%, specificity of 65.3%, whereas cutoff value of CA 15-3 was >21.24 (U/ml) with a sensitivity of 56.7%, specificity of 74.5%. For CEA at cutoff value >0.99 (ng/ml) the sensitivity and specificity were 96.7 % and 62.7% respectively. AUC for HE4, CA15-3 and CEA were 0.725, 0.644 and 0.857 respectively.

**Conclusion:**

Our study demonstrated that serum levels of HE4 were significantly higher in malignant group compared to benign and control groups. There is no significant difference between HE4 levels between benign and control groups. These results indicate that HE4 appears as a useful and highly specific biomarker for breast cancer, which can differentiate between malignant and benign tumors.

## Introduction

Breast cancer is the most common cancer in women worldwide representing nearly a quarter of all cancers with an estimated 1.67 million new cancer cases diagnosed in 2012. Women from less developed regions have slightly more number of cases compared to more developed regions (Ferlay et al., 2015). Breast cancer has ranked number one cancer among Indian women with age adjusted rate as high as 25.8 per 100,000 women and mortality 12.7 per 100,000 women (Malvia et al., 2017). Changes in lifestyle increased risk-factor profile. Despite the rising incidence of breast cancer, significant improvement of survival in recent years is due to the progress of research at molecular and biological levels of breast cancer. However, it is essential to identify reliable prognostic factors to guide decision making during the treatment of breast cancer. There is lack of specific and sensitive serum biomarkers for detection and disease progression. Along with tumor size, grade, lymph node status, invasion, molecular markers including hormone receptor status and human epidermal growth factor receptor 2 (HER2) expression (Galgano et al., 2006), have an important role in screening, early diagnosis of recurrence, and treatment of many malignancies (Hellström et al., 2003; Bingle et al., 2006).

In carcinoma of the breast, the most commonly used serum tumor marker is cancer antigen 15-3 (CA 15-3); however, its sensitivity and specificity are inadequate (Kamei et al., 2010; O’Neal et al., 2013). The lack of sensitivity and specificity of the present diagnostic parameters have led to the search for newer biomarkers to add to the present panel to identify the disease earlier and halt its progression.

Human epididymal protein 4 (HE4) is a secretory protein initially identified in epithelial cells of the human epididymis (Donepudi et al., 2014). Expression of HE4 has been demonstrated in numerous types of normal human tissues, particularly in the epithelium of the respiratory and genitourinary tracts of men and women, and increased HE4 expression has been demonstrated in a range of malignant neoplasms, particularly those of gynecological, pulmonary, and gastrointestinal origin (Galgano et al., 2006; Geng et al., 2015; Ideo et al., 2015). It has been recently reported that HE4 is also expressed in ductal carcinoma of the breast tissue (Geng et al., 2015); however, its serum expression levels and their diagnostic and prognostic potential in breast cancer remain to be elucidated. HE4 is closely associated with lymph node metastases. These findings suggest that HE4 is a possible predictive marker of lymph node metastasis and has a critical role in its recurrence (Hellström et al., 2003; Bingle et al., 2006).

So we aimed to estimate the levels of HE4 in established breast cancer, benign breast lump cases and healthy controls and to evaluate the clinical eligibility of HE4 as a potential tumor marker.

## Materials and Methods

A cross-sectional case-control study was conducted in departments of Biochemistry, Pathology and Surgical Oncology of a tertiary care hospital at Hyderabad, India from June to December 2019. Based on the alpha error at 0.05 and power of 0.8, standard deviation of group 1 and 2 as 26.19 and 2.19 respectively, difference of means as 14.89 and with ratio of sample sizes in group 1 to 2 as 3.27 with p value <0.05 from previous study (Gunduz et al., 2016) sample size calculated was 27 cases 09 and controls (MedCalc Software., 2019). Female patients with a newly diagnosed breast lump were recruited and grouped into malignant (n=30) and benign (n=30) groups after confirmation with biopsy. Thirty age matched controls were selected from healthy women volunteers. Patients with a previous history of breast or other cancers including that of endometrium, ovary etc were not included in the study. The study was approved by Institutional Ethical Committee (EC/NIMS/1990/2017). Informed consent was taken from all the participants. Samples from the cases were collected preoperatively. All the breast lump cases have undergone Chest X-ray, Bilateral mammography, FNAC and Core needle biopsy. Serum CA15-3, CEA and HE4 levels were measured on Roche Cobas e411 by electro-chemiluminescence immunoassay (diagnostics.roche.com). 

*Statistical methods*

Statistical analysis was performed using MedCalc^®^ Statistical Software version 19.6.1 (MedCalc Software Ltd, 2019). Distribution of normality was established by the ShapiroWilk normality test. KruskalWallis test with the post hoc Dunn’s multiple comparison method was used to determine the statistical significance across the three groups (Malignant, Benign and Control). Receiver operating characteristics (ROC) curves were used to evaluate the diagnostic utility of HE4, CA15-3 and CEA as estimated by the area under the curve (AUC), sensitivity, specificity, positive predictive value and negative predictive value. P < 0.05 is considered as statistically significant.

## Results

Out of the total 90 women included in this study 63 were premenopausal women (30 in benign, 13 in malignant and 20 in the control group) and 27 postmenopausal women (17 in malignant and 10 in control groups). In the benign group, the most represented histological type was Fibroadenoma (77.67%), followed by benign phyllodes tumour (13.33%) and Intra-ductal papilloma (10%). In malignant group, the most common was invasive ductal carcinoma (IDC) (66.67%) followed by IDC with extensive ductal carcinoma in Situ DCIS (13.33%). 

Median age of cancer patients was 54 yrs (31 to 72 yrs). Median age in cases of benign group was 34 years (18 to 45 yrs). The Median age in malignant cases was significantly higher than in benign group (Table 1). Most common stage at which breast cancer patients presented was Stage IIIB. 

A significant difference was noted in HE-4 (pmol/L) levels [Median(IQR)] of controls 52.3(50.6- 63.3), malignant 62.4(52.6- 73.7) and benign 49.34(39.8-57.4) groups with p value of <0.0009 (Table 1). Median levels revealed significant difference in HE4 levels of malignant group when compared with controls (p=0.0465) and with benign group (p=0.0004). But there was no significant difference of HE4 between benign and control groups.

Similarly significant differences in CEA levels (ng/ml) were also noted between malignant [2.067(1.5 – 3.6)] and control [0.21(0.21 – 0.23)] groups (p<0.0001), malignant and benign [1.37(0.8 -1.8)] groups (p=0.0012), benign and control groups (p=<0.0001). Whereas CA15-3 did not show any significant difference among the three groups (Table 1). 

**Figure 1:**
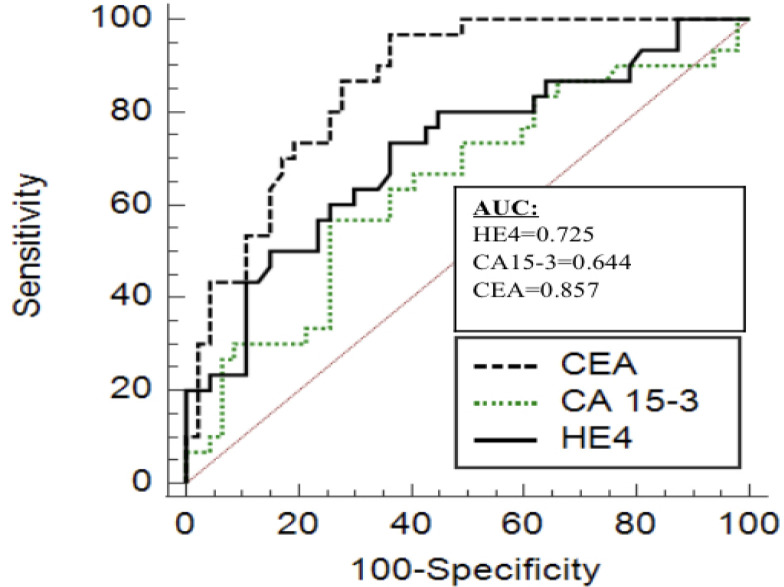
Comparison of AUC of HE4, CA 15-3 and CEA in Breast Cancer. AUC, Area under curve; HE-4, Human Epididymis protein-4; CEA, Carcinoembryonic antigen; CA 15-3, carbohydrate antigen 15-3

To assess the diagnostic performance of various biomarkers, ROC analysis was done. HE4 had a sensitivity of 73.3% and specificity of 65.3% with AUC of 0.725 at a cutoff of >54.5pmol/L for diagnosis of breast cancer. CEA had a sensitivity of 96.7% and specificity of 62.7% with AUC of 0.857 at a cut-off of 0.99ng/ml. CA15-3 showed a sensitivity of 56.7% and specificity of 74.5% with AUC of 0.644 at a cut-off level of 21.24U/ml (Table 2 and Figure 1). 

**Figure 2:**
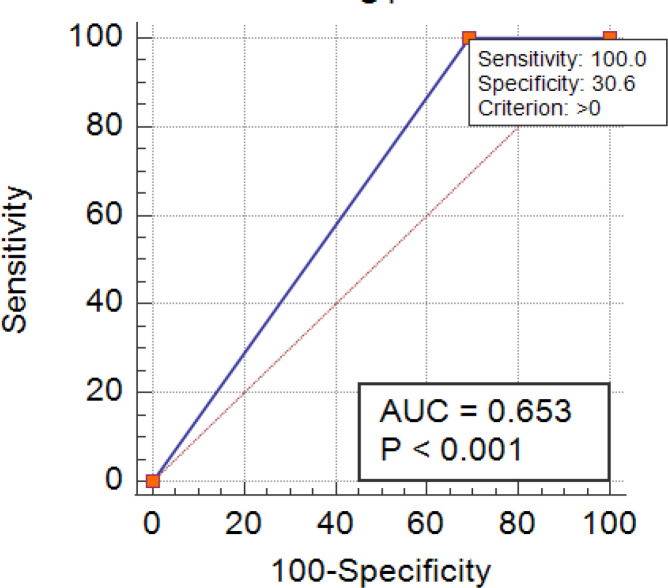
Combined AUC of HE4, CA 15-3 and CEA in Breast Cancer Patients. AUC, Area under curve; HE-4, Human Epididymis protein-4; CEA, Carcinoembryonic antigen; CA 15-3, carbohydrate antigen 15-3

The combination of HE4, CEA and CA15-3 showed sensitivity and specificity of 100% and 30.6% respectively with AUC 0.653 (Figure 2). Serum HE4 has not shown any significant correlation with either CEA (r=0.056, p=0.63) or CA15-3 (r=0.036, p=0.75).

**Table 1:** Biomarkers in Control Breast Tumor Groups

Variable	Control Group (n=30)	Benign Group (n=30)	Malignant group (n=30)	p value
Age (years)	43 (28-62)	30 (18-45)	54 (31-72)	<0.001*
HE4 (pmol/l)	52.3 (50.6-63.3)	49.34 (39.8-57.4)	62.4 (52.6-73.7)^a,b^	< 0.0009*
CA 15-3 (U/ml)	15.4 (11.2 - 21.9)	16.9 (13.4-24.1)	21.8 (15.1-29.1)	0.19
CEA (ng/ml)	0.21(0.21 - 0.23)	1.37 (0.8-1.8)^c^	2.067 (1.5- 3.6)^d,e^	0.000001*

HE-4, Human Epididymis protein-4; CEA, Carcinoembryonic antigen; CA 15-3, carbohydrate antigen 15-3; *p <0.05 is significant;“a”, significant difference in HE4 levels between malignant and control groups; “b”, significant difference in HE4 levels between malignant and benign group; “c”, significant difference in CEA levels between benign and control groups; “d”, significant difference in CEA levels between malignant and control groups; “e”, significant difference in CEA levels between malignant and benign groups (Dunn’s post-hoc multiple comparision analysis).

**Table 2:** Diagnostic Efficacy of the Biomarkers in Breast Tumors

Biomarker	Cut-off values	Sensitivity%	Specificity%	PPV%	NPV%	AUC
HE4	>54.5 pmol/l	73.3	65.3	56.4	80	0.725
CA15-3	>21.24 U/ml	56.7	74.5	58.7	72.9	0.644
CEA	>0.99 ng/ml	96.7	62.7	60.4	97.0	0.857

HE-4, Human Epididymis protein-4; CEA, Carcinoembryonic antigen; CA 15-3, carbohydrate antigen 15-3; PPV, Positive predictive value; NPV, negative predictive value; AUC, Area under curve.

## Discussion

Breast cancer is a vast group of diseases with varied clinical presentation and pathological characteristics. The treatment course, recurrence and prognosis are affected by the biological features of the tumor and the stage at diagnosis. The effort with the novel biomarkers for breast cancer diagnosis is to improve the accuracy of the detection and assess the severity of the malignancy at earliest possible stage.

Serum markers like BR27.29 (CA 27.29), CA15-3, mucin like carcinoma associated antigen, CA 549, and CEA with limited sensitivity and specificity have been investigated; however, none of these markers have reached the standards required for clinical practice (Bingle et al., 2006).

Serum HE4, also known as whey acidic four disulfide core domain protein 2 (WFDC2), encoded by the WFDC2 gene has been introduced for the routine diagnostics of ovarian cancer. HE4 was first described in normal tissues such as the epithelium of epididymis, and the bronchial epithelium in the proximal respiratory tract (Bingle et al., 2002).

Limited data describing HE4 as a diagnostic and prognostic marker in breast cancer especially in that of Indian population is available. The prospective use of HE4 as a tumor marker particularly of gynecological, pulmonary, and gastrointestinal origin has been established by a number of studies (Hellström et al., 2003; Geng et al., 2015). The serological detection of HE4 has been shown to have increased sensitivity and specificity in the detection of ovarian cancer compared with CA 125, which is the current gold standard serum biomarker for ovarian carcinoma (Ferraro et al., 2013; Zhen et al., 2014).

While all these previous literature indicated the importance of HE4 in ovarian cancer, the purpose of this study was to evaluate the clinical utility of HE4 as a potential tumor marker in breast cancer patients.

Our observations have shown significant difference in serum HE4 levels among malignant, benign and controls. Post-hoc analysis revealed significant difference when malignant group was compared to benign (p=0.0004) and controls (p=0.0465). No difference was seen between benign cases and controls. However no difference was found in CA15-3 levels between any groups. Even though CEA levels were significantly different among all the groups with better AUC, it is known to increase in many cancers which make it a general cancer marker not specific to any particular cancer. Thus our findings are intersting in that out of three markers studied, CA15-3 didn’t show any significant increase in malignant group and CEA showed increase in both malignant and benign groups whereas HE4 increased in only malignant group but not in benign or control groups.

 There appears to be no significant correlation between HE4, CA 15-3 (r=0.036; p=0.75) and CEA (r=0.056; p=0.63) in our study population. The cutoff value of HE4 levels for predicting breast cancer is >54.5 pmol/l with a sensitivity of 73.3%, specificity 65.3%, positive predictive value 56.4%, negative predictive value 80% and AUC of 0.725. These findings indicate that HE4 may be used as a predictive marker for breast carcinoma. Galgano et al., (2006) reported the mRNA and protein expression of HE4 in normal and malignant tissues. In addition, Kamei et al., (2010) found that the increased expression of HE4 in breast cancer tissues correlated with lymph node invasion and was a possible predictive factor of breast cancer recurrence.

Our observations indicate that HE4 is a significant biomarker associated with malignant breast cancer. To the best of our knowledge, the serum levels of HE4 in breast cancer patients, and their diagnostic, prognostic potential have not been investigated in general and in Indian population specifically. In the current study, the serum levels of HE4 in patients diagnosed with breast cancer were assessed prior to any form of treatment and compared with those in healthy individuals and benign breast lump cases. The serum levels of HE4 were significantly increased in patients with breast malignancy compared with those in benign cases and healthy controls. The sensitivity and the specificity of serum HE4 was reasonable in distinguishing breast cancer patients from benign and healthy controls. These findings indicate that HE4 may be used as a predictive marker for breast carcinoma.

In the present study, serum HE4 levels based on menopausal status, stages of cancer, hormone receptor status were not statistically significant similar to Kamei et al., (2010) and Gunduz et al., ( 2016). Multivariate analysis did not show any significant positive correlation of HE4 serum levels with histological grade and clinical stage in breast cancer patients.

In conclusion, the significant elevation of HE4, an ovarian cancer marker, in malignant breast tumor patients and its non elevation in benign breast tumor patients makes it an interesting and important biomarker in the evaluation of breast tumors, in addition to the current markers. Further exploration of this marker in other cancers will help to bring out its specificity more clearly.

## Author Contribution Statement

Study conception and design: Dr.KSS Sai Baba, Dr. Noorjahan; data collection: Dr. M.A.Rehman, Dr. Pradeep and Dr.Maira; analysis and interpretation of results:, Dr.Pradeep, Dr.Maira Dr.Shantveer and Dr.GSN Raju; draft manuscript preparation: Dr.KSS Sai Baba, Dr. Noorjahan, Dr. M.A.Rehman. All authors reviewed the results and approved the final version of the manuscript.
